# Development of a real-time PCR assay to detect the single nucleotide polymorphism causing Warmblood Fragile Foal Syndrome

**DOI:** 10.1371/journal.pone.0259316

**Published:** 2021-11-08

**Authors:** Sharon Flanagan, Áine Rowe, Vivienne Duggan, Erin Markle, Maureen O’Brien, Gerald Barry

**Affiliations:** School of Veterinary Medicine, University College Dublin, Belfield, Dublin, Ireland; King Abdulaziz University Hospital, SAUDI ARABIA

## Abstract

Warmblood Fragile Foal syndrome (WFFS) is an autosomal recessive condition that affects the maturation of collagen in affected foals. Foals affected with the disease typically die or are euthanised shortly after birth. WFFS is caused by a single nucleotide change at position 2032 of the equine PLOD1 gene, causing an impairment of the wild-type enzyme. A commercial test for the causative genetic mutation is currently available from companies operating under licence from Cornell University but it has limitations. This test requires amplification of a region of the PLOD1 gene encompassing the site of interest, followed by Sanger sequencing of that region and computational analysis. We describe here the development of an alternative, real-time PCR based assay that rapidly and reliably differentiates between the wild-type and WFFS associated nucleotides without the need for sequencing, thus increasing the potential for high throughput analysis of large numbers of samples in a cost-effective manner.

## Introduction

**Fig 1:**
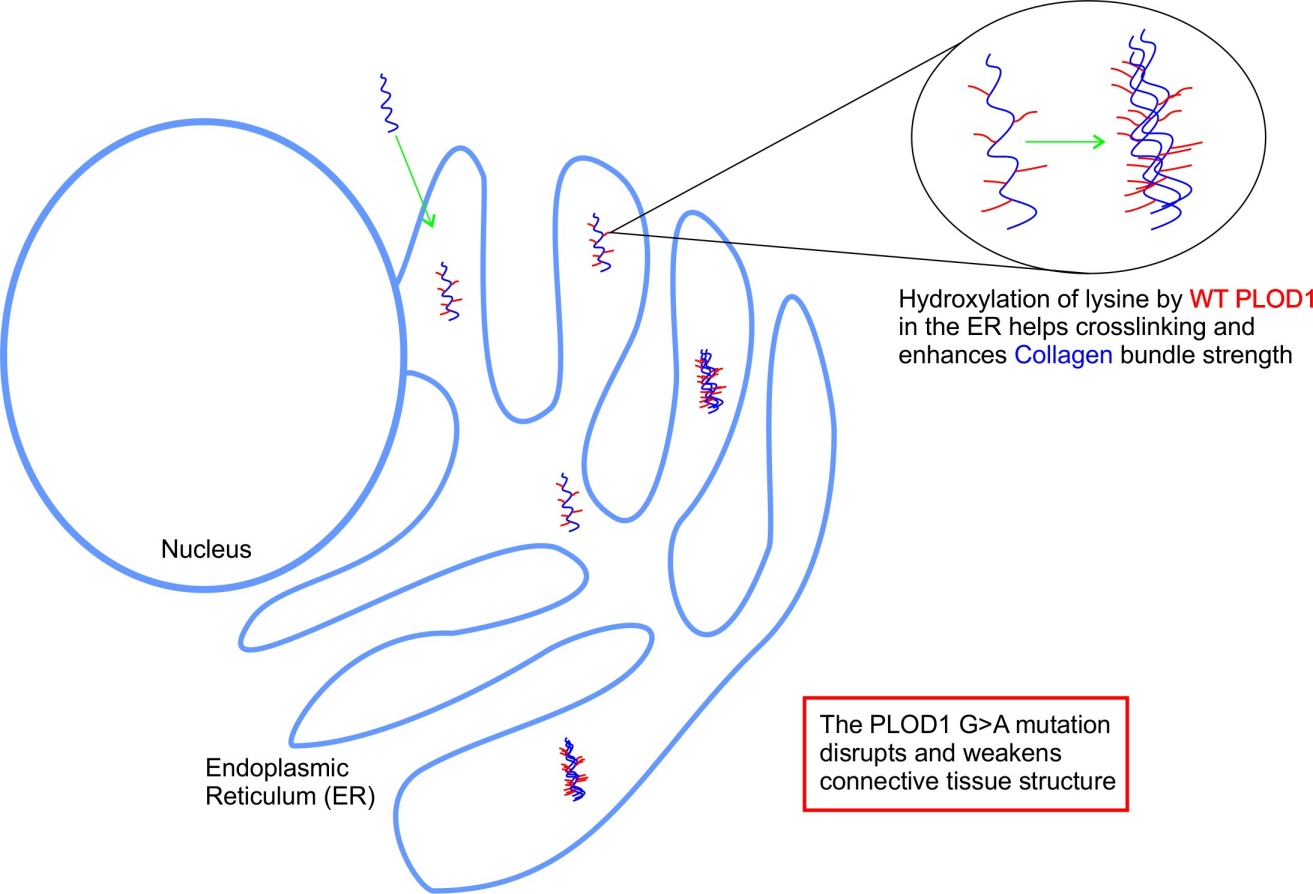
Hydroxylated lysines and prolines become glycosylated, fibres come together to form triple helixes and get transported out of the ER. Hydroxylation (red) is crucial for the crosslinking of fibres and improving the strength of the collagen bundles. This does not happen properly if hydroxylation is faulty as is the case in WFFS.

Warmblood fragile foal syndrome (WFFS) is an autosomal recessive genetic condition affecting the connective tissue of Warmblood foals [1]. The disorder is caused by a mutation in procollagen-lysine, 2-oxoglutarate 5-dioxygenase1 (PLOD1) gene [2, 3]. The PLOD1 gene encodes for the enzyme lysyl hydroxylase, which is responsible for converting lysine to hydroxylysine through hydroxylation. The hydroxyl group of hydroxylysine binds to specific galactose mono- and disaccharides that improve collagen structural strength (Fig 1) [4–6]. Affected foals have a homozygous non-synonymous G>A mutation at nucleotide position 2032 of the PLOD1 gene, resulting in a glycine to arginine substitution at amino acid position 678 [2].

Warmblood Fragile Foal Syndrome is associated with a variety of clinical signs including excessive skin fragility, open skin wounds, flexural deformities, deformities of the spinal canal and perforating lesions of the aorta. Hyper-extensibility of joints, often resulting in dystocia, has frequently been reported. Clinical signs are incompatible with extra uterine life and foals die or are euthanised within hours of birth [1, 7, 8].

As the heterozygous genotype has not been associated with clinical abnormalities, the presence of the mutation in adult horses is often not detected until the birth of an affected foal, which can result in considerable economic losses for the breeder. Genetic testing of breeding mares and stallions for the mutation is vital to prevent the unknown mating of WFFS carriers, thus eliminating the risk of the birth of unviable foals.

In previous studies carried out in a number of different locations across the world, prevalence of heterozygous carriers has ranged from less than 5% to 17% [8–11]. Further prevalence studies are required in order to determine the true prevalence of the mutation in the equine population globally. Although typically associated with Warmblood breeds, the mutation has also been identified in non-Warmblood breeds, including the Thoroughbred [7, 9, 10], Quarter Horse [3] and American Sport Pony [9], highlighting the importance of genetic testing for the mutation, in clinically suspicious cases, in non-Warmblood breeds as well as Warmblood breeds.

Commercial testing of horses can be costly, particularly the testing of large numbers, as is the case when performing prevalence studies. Affordable and readily accessible genetic testing for the WFFS causative mutation is important for the equine industry. Increased use of genetic testing increases our knowledge of this disorder and allows breeders to make more informed breeding decisions. With this in mind, we have developed a quantitative polymerase chain reaction (qPCR) based assay, as an alternative to the two step PCR and Sanger Sequencing assay currently commercially provided by laboratories working under licence to Cornell University [2].

## Materials and methods

The protocol described in this peer-reviewed article is published on protocols.io, https://dx.doi.org/10.17504/protocols.io.bw4fpgtn, and is included for printing as S1 File with this article.

### Collection of samples

Hair samples were collected with consent from a variety of horse and pony breeds. The hair was manually pulled from the mane to ensure as little discomfort to the horse as possible. The hair was sealed in a biosafety bag and stored at room temperature before testing. The study was conducted under ethical exemption from the University College Dublin Animal Research Ethics Committee: (AREC-E-18-47-Duggan).

### DNA extraction

The Qiagen DNA Blood and Tissue kit was used, and the extraction protocol was adapted from the manufacturer’s instructions. Hair strands (10 per extraction) were cut at a maximum length of 1 cm, including the follicular tag. The hair was placed into a 1.5 ml Rnase, DNase-free micro-centrifuge tube followed by 300 μl of ATL Buffer, 20 μl of Proteinase K and 20ul of a 1 M DTT solution. The sample was incubated at 56°C with occasional vortexing until the hair was completely lysed and could not be seen anymore. After incubation, the sample was vortexed for 15 seconds to ensure complete homogenisation before 300 μl AL Buffer was added and it was again briefly vortexed. This was followed by the addition of 300 μl of ethanol (molecular grade, > 99.5% pure) with brief vortexing again to ensure complete mixing. The sample mixture was pipetted into a DNeasy mini spin column and centrifuged at room temperature at 6000 x g for 1 minute. The flow through was discarded and two wash steps were carried out on the column using the same centrifugation conditions as before–firstly 500 μl of AW1 Buffer was added and the column was centrifuged, followed by 500 μl of buffer AW2 and centrifugation. After each centrifugation, the flow-through was discarded. An extra centrifugation step (dry) was added to remove any excess ethanol before the extracted DNA was eluted into a clean RNase, DNase-free 1.5ml microcentrifuge tube using 100 μl of elution buffer AE. For elution, the buffer was added to the column, allowed to incubate for 1 minute at room temperature and then centrifuged before being re-added to the column, re-incubated for 1 minute and then centrifuged through. This second incubation increased the yield by approximately 15%. Sample concentration and purity was determined using a Nanodrop One instrument before proceeding to the next step.

### Plasmid controls

To provide controls for the PCR and for initial optimisation of the assays, regions of the PLOD1 gene that included the region of interest were synthesized commercially and inserted into a pEX-A128 plasmid vector (Eurofins Genomics). These plasmids were prepared in standard DH5-alpha *E-coli* using a Qiagen mini-prep kit. This method produced control plasmids that were approximately 60 ng/μl in concentration. Individually the plasmids were used as the homozygous controls, and when mixed in equal concentrations together they acted as the heterozygous control.

### Real-time PCR design

The real-time PCR assay was based on a TaqMan SNP Genotyping assay (Thermo Fisher Scientific). Two primers were designed to amplify the region of equine PLOD1 that contained the SNP of interest. The primers were designed to not amplify human PLOD1, to avoid any potential contamination issues. Two allele specific probes were also designed, that would bind to the exact region where the SNP would be present. One probe sequence matched the wild-type sequence and the other recognised the mutant version (see Table 3). Each probe had a different fluorescent dye (FAM–mutant and VIC–wild-type) bound to it along with a quencher (TAMRA). The principle of the assay is that during the PCR cycle, if the wild-type sequence is present the wild-type probe will bind, DNA polymerase will interact with it during amplification and knock off the fluorescent dye, releasing it from the quencher and allowing a signal to be produced and measured. In contrast, if the mutant sequence is present, the mutant specific probe will bind, and its specific fluorescent signal will be produced.

### Real-time PCR assay conditions

Each PCR was set up in a 96 well plate as outlined in Table 1 and centrifuged briefly to eliminate air bubbles and to ensure the reaction mixture was in the bottom of the wells. Duplicate plasmid controls for homozygous mutant, homozygous wild-type and heterozygous sequences were included on each run.

**Table 1:** The component volumes for the real-time PCR with dual labelled TaqMan probes.

Component	Volume per 20 μl reaction
TaqPath ProAmp Master Mix	10 μl
TaqMan Genotyping Assay	0.5 μl
Genomic DNA or NTC	5.0 μl
Nuclease- Free water	4.5 μl
Total Volume	20 μl

The plate was sealed with an optical adhesive cover and placed in the Applied Biosystems 7500 Real-Time PCR system which functioned under the reaction conditions detailed in Table 2. The sequence of the primers and probes are detailed in Table 3; probe sequences were designed with the help of Thermo Fisher Scientific to create a non-human custom TaqMan SNP Genotyping Assay. A detailed step by step protocol is available in S1 File.

**Table 2:** The conditions for the real-time PCR assay with dual labelled TaqMan probes.

PCR Step	Temperature	Time	Cycles
Pre–Read	60°C	30 seconds	Hold
Initial denaturation / Enzyme Inactivation	95°C	5 minutes	
Denature	95°C	15 seconds	40
Anneal / Extend	60°C	60 seconds	
Post–Read	60°C	30 seconds	Hold

**Table 3:** Sequences for the primers and probes used.

Component	Sequence
SNP Genotyping assay Forward Primer	TCCTGTTGGGAAACTGACACTTC
SNP Genotyping assay Reverse Primer	TCGGATGGAGCAGTTGTAACG
FAM Probe	ACAGCCCCTGCCCTG
VIC Probe	CAGCCCCCGCCCTG

### Validation

In Ireland one commercial company performs the genetic test for WFFS and this company was used to validate the newly developed PCR based assay being assessed here. Hair samples were sent to the company for analysis in an anonymous format and results from them were taken as the current ‘gold standard’.

## Results

### Optimisation of the PCR

**Fig 2:**
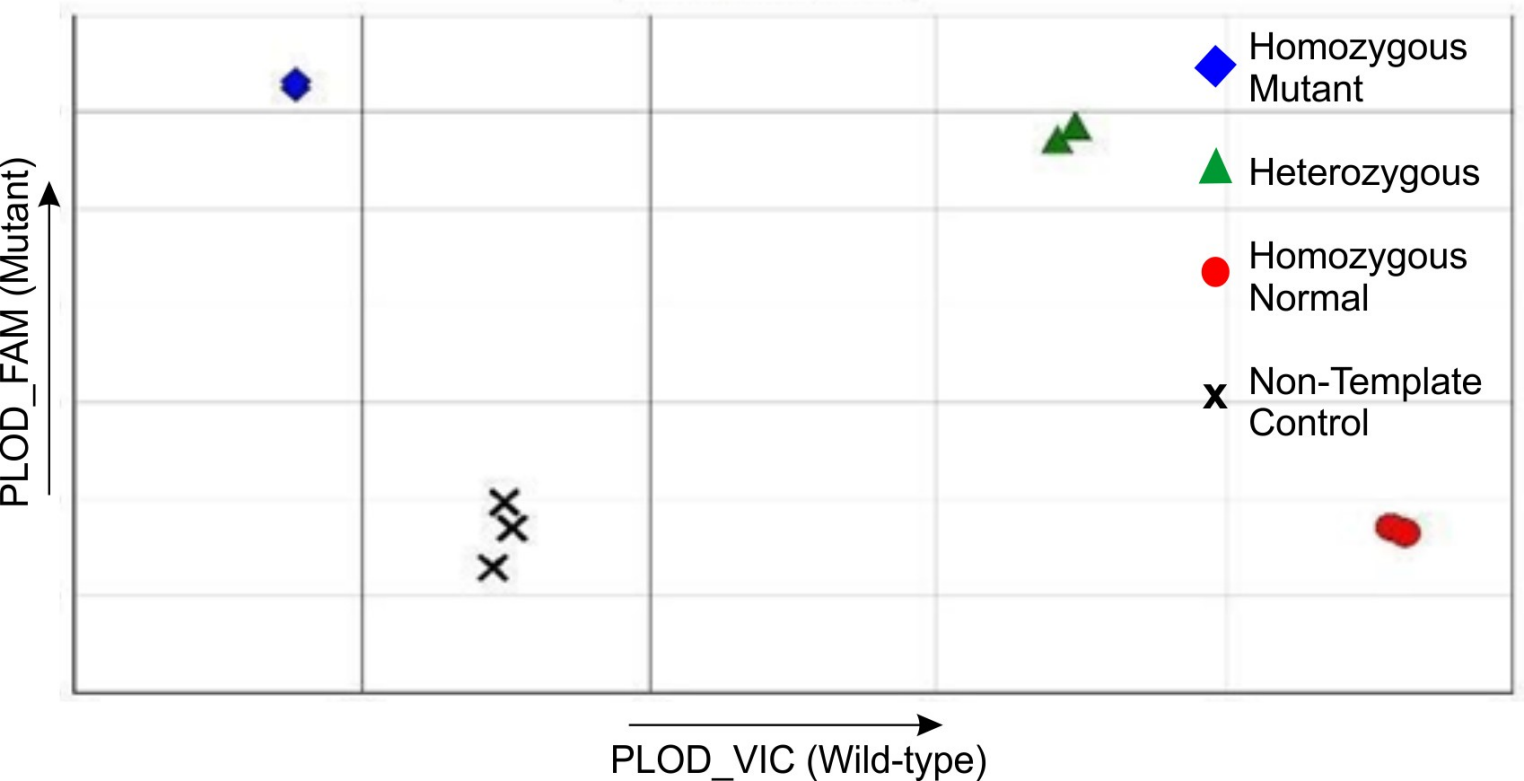
Example of a PCR with the three genotypic controls, showing how the SNPs can be differentiated.

PCR conditions were initially optimised using the control plasmids. Varying concentrations of each plasmid were used to individually establish optimal conditions for both the wildtype and mutant sequences. The optimal concentration for each plasmid was 6 ng/μl. Following this, the plasmids were mixed to create a heterozygous situation as would be seen in carriers. Equal concentrations of each plasmid were added to the reaction mix, while maintaining the same volume, and the real–time PCR reaction was shown to effectively distinguish between them, despite only a single nucleotide difference being present (Fig 2).

Following optimization using plasmids, DNA was extracted from 20 hair samples, each from a different animal, and the same real-time PCR assay was carried out. Different concentrations of samples were tested and 10 ng/μl was found to be optimal. Samples were run in duplicate each time and repeated at least three times to confirm the robustness of the assay, with a consistent result achieved each time. The assay reliably identified whether a sample was homozygous wild-type or heterozygous. No homozygous mutants from hair samples were identified.

### Validation of results

**Fig 3:**
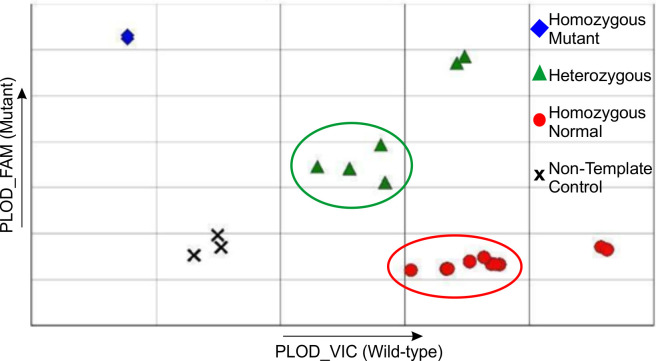
Example result from the newly developed PCR. Samples from horses are circled, control samples are those that are not circled.

A total of 18 samples were tested by an external commercial test and compared to the newly developed PCR (example in Fig 3). All 18 samples results matched using both assays, 15 samples were homozygous normal on both tests, 3 samples were heterozygous on both tests.

## Discussion

WFFS is a fatal condition for horses that may be economically and emotionally devastating for the owner involved. The mating of heterozygous carriers creates a 1 in 4 chance of creating a homozygous mutant. Independent of the potential positives or negatives, increased understanding of the genotypic status of horses allows informed choices to be made and should be seen as a positive and progressive step towards improving equine health. There is a need to carry out large scale prevalence studies and to encourage owners to screen their animals for this mutation, so inappropriate mating can be avoided. The newly developed WFFS assay reported here is an improvement on what is currently available. It is an efficient, reliable, and rapid test that allows high-throughput analysis of samples thus reducing the associated costs. The new assay is faster than any current test being used: From DNA extraction to PCR result is approximately 6 hours while in our experience, the Sanger sequencing route can take approximately 3 days, while the commercial company we used (only one available in Ireland) took 2 weeks to return results. In terms of cost, both the new qPCR method and a Sanger sequencing route are approximately comparable (excluding start up hardware costs), while the cost of testing a batch of 40 samples commercially was approximately 4 times the cost per sample compared to the qPCR assay described here, again excluding start up hardware costs. There is potential to decrease the price of the real-time PCR based assay further if sample numbers are increased or steps could be automated. In summary, this newly developed assay is an excellent alternative to currently used options and we recommend its use in WFFS associated mutation prevalence studies that are planned in the future.

## Supporting information

S1 File (PDF)
