# Supplementary material for: Development of a real-time PCR assay to detect the single nucleotide polymorphism causing Warmblood Fragile Foal Syndrome
Source: PLoS One. 2021 Nov 8;16(11):e0259316. doi: 10.1371/journal.pone.0259316 (PMC8575260; doi:10.1371/journal.pone.0259316)
Supplement: S1 File — (PDF) [file pone.0259316.s001.pdf]

Aug 24, 2021

PCR protocol to identify an equine mutation associated with Warmblood Fragile Foal Syndrome(WFFS)

PLOS One Peer-reviewed method

gerald.barry [1], Sharon Flanagan[1], Áine Rowe[1], Vivienne Duggan[1], Erin Markle[1], Maureen O'Brien[1]

[1]University College Dublin

1 Works for me Share

dx.doi.org/10.17504/protocols.io.bw4fpgtn

GB Lab

gerald.barry

DISCLAIMER

DISCLAIMER – FOR INFORMATIONAL PURPOSES ONLY; USE AT YOUR OWN RISK

The protocol content here is for informational purposes only and does not constitute legal, medical, clinical, or safety advice, or otherwise; content added to protocols.io is not peer reviewed and may not have undergone a formal approval of any kind. Information presented in this protocol should not substitute for independent professional judgment, advice, diagnosis, or treatment. Any action you take or refrain from taking using or relying upon the information presented here is strictly at your own risk. You agree that neither the Company nor any of the authors, contributors, administrators, or anyone else associated with protocols.io, can be held responsible for your use of the information contained in or linked to this protocol or any of our Sites/Apps and Services.

Citation: gerald.barry , Sharon Flanagan, ÃÂine Rowe, Vivienne Duggan, Erin Markle, Maureen OÃ¢ÂÂBrien PCR protocol to identify an equine mutation associated with Warmblood Fragile Foal Syndrome(WFFS) https://dx.doi.org/10.17504/protocols.io.bw4fpgtn

This is an open access protocol distributed under the terms of the **Creative Commons Attribution License** (https://creativecommons.org/licenses/by/4.0/), which permits unrestricted use, distribution, and reproduction in any medium,

ABSTRACT

Warmblood Fragile Foal syndrome (WFFS) is an autosomal recessive condition that affects the maturation of collagen in affected foals. Foals affected with the disease typically die or are euthanised shortly after birth. WFFS is caused by a single nucleotide change at position 2032 of the equine PLOD1 gene, causing an impairment of the wild-type enzyme. A commercial test for the causative genetic mutation is currently available from companies operating under licence from Cornell University but it has limitations. This test requires amplification of a region of the PLOD1 gene encompassing the site of interest, followed by Sanger sequencing of that region and computational analysis. We present here the development of an alternative, real-time PCR based assay that rapidly and reliably differentiates between the wild-type and WFFS associated nucleotides without the need for sequencing, thus increasing the potential for high throughput analysis of large numbers of samples in a cost-effective manner.

DOI

dx.doi.org/10.17504/protocols.io.bw4fpgtn

EXTERNAL LINK

https://doi.org/10.1186/s13620-021-00206-1

PROTOCOL CITATION

gerald.barry , Sharon Flanagan, Áine Rowe, Vivienne Duggan, Erin Markle, Maureen O'Brien 2021. PCR protocol to identify an equine mutation associated with Warmblood Fragile Foal Syndrome(WFFS). **protocols.io** https://dx.doi.org/10.17504/protocols.io.bw4fpgtn

MANUSCRIPT CITATION please remember to cite the following publication along with this protocol

Rowe

LICENSE

This is an open access protocol distributed under the terms of the Creative Commons Attribution License, which permits unrestricted use, distribution, and reproduction in any medium, provided the original author and source are credited

CREATED

Aug 03, 2021

LAST MODIFIED

Aug 24, 2021

PROTOCOL INTEGER ID

52071

Citation: gerald.barry , Sharon Flanagan, ÃÂine Rowe, Vivienne Duggan, Erin Markle, Maureen OÃ¢ÂÂBrien PCR protocol to identify an equine mutation associated with Warmblood Fragile Foal Syndrome(WFFS) https://dx.doi.org/10.17504/protocols.io.bw4fpgtn

This is an open access protocol distributed under the terms of the **Creative Commons Attribution License** (https://creativecommons.org/licenses/by/4.0/), which permits unrestricted use, distribution, and reproduction in any medium,

DISCLAIMER:

DISCLAIMER – FOR INFORMATIONAL PURPOSES ONLY; USE AT YOUR OWN RISK

The protocol content here is for informational purposes only and does not constitute legal, medical, clinical, or safety advice, or otherwise; content added to protocols.io is not peer reviewed and may not have undergone a formal approval of any kind. Information presented in this protocol should not substitute for independent professional judgment, advice, diagnosis, or treatment. Any action you take or refrain from taking using or relying upon the information presented here is strictly at your own risk. You agree that neither the Company nor any of the authors, contributors, administrators, or anyone else associated with protocols.io, can be held responsible for your use of the information contained in or linked to this protocol or any of our Sites/Apps and Services.

DNA extraction

1 Hair strands (10 per extraction) are cut at a maximum length of 1 cm, including the follicular tag.

2 The hair is placed into a 1.5 ml Rnase, DNase-free micro-centrifuge tube followed by 300 µl of ATL Buffer, 20 µl of Proteinase K and 20ul of a 1 M DTT solution.

3 The sample is incubated at 56oC with occasional vortexing until the hair is completely lysed and cannot be seen anymore.

4 After incubation, the sample is vortexed for 15 seconds to ensure complete homogenisation.

5 300µl AL Buffer is added and the sample is again briefly vortexed.

6 300µl of ethanol (molecular grade, > 99.5% pure) is added with brief vortexing again to ensure complete mixing.

7 The sample mixture is pipetted into a DNeasy mini spin column and centrifuged at room temperature at 6000 x g for 1 minute. The flow through is discarded

Citation: gerald.barry , Sharon Flanagan, ÃÂine Rowe, Vivienne Duggan, Erin Markle, Maureen OÃ¢ÂÂBrien PCR protocol to identify an equine mutation associated with Warmblood Fragile Foal Syndrome(WFFS) https://dx.doi.org/10.17504/protocols.io.bw4fpgtn

This is an open access protocol distributed under the terms of the Creative Commons Attribution License (https://creativecommons.org/licenses/by/4.0/), which permits unrestricted use, distribution, and reproduction in any medium,

8 500µl of AW1 Buffer is added and the column is centrifuged at room temperature at 6000 x g for 1 minute and the flow through discarded.

9 500µl of AW2 Buffer is added and the column is centrifuged at room temperature at 6000 x g for 1 minute and the flow through discarded.

10 The samples are centrifuged again at room temperature at 6000 x g for 1 minute to remove any excess ethanol before the extracted DNA was eluted into a clean RNase/DNase-free tube.

11 For elution, 100 µl of buffer AE was added to the column, allowed to incubate for 1 minute at room temperature and then centrifuged at room temperature at 6000 x g for 1 minute.

12 The sample is re-added to the column, re-incubated for 1 minute and then centrifuged at room temperature at 6000 x g for 1 minute.

RT-PCR

13 Each PCR reaction is set up as per the table below in a 96 well plate.

**Component**	**Volume per 20 µl reaction**
TaqPath ProAmp Master Mix	10 µl
TaqMan Genotyping Assay	0.5 µl
Genomic DNA or NTC	5.0 µl
Nuclease- Free water	4.5 µl
Total Volume	20 µl

14 The well plate is sealed with PCR compatible optical adhesive film and centrifuged at room temperature at 220 x g for 1 minute.

15
1. The PCR running conditions are set up as per the table below. We used an Applied Biosystems 7500 RT-PCR machine.

Citation: gerald.barry , Sharon Flanagan, ÃÂine Rowe, Vivienne Duggan, Erin Markle, Maureen OÃ¢ÂÂBrien PCR protocol to identify an equine mutation associated with Warmblood Fragile Foal Syndrome(WFFS) https://dx.doi.org/10.17504/protocols.io.bw4fpgtn

This is an open access protocol distributed under the terms of the **Creative Commons Attribution License** (https://creativecommons.org/licenses/by/4.0/), which permits unrestricted use, distribution, and reproduction in any medium,

PCR Step	Temperature	Time	Cycles
Pre – Read	60oC	30 seconds	Hold
Initial denaturation / Enzyme Inactivation	95oC	5 minutes	
Denature	95oC	15 seconds	40
Anneal / Extend	60oC	60 seconds	
Post – Read	60oC	30 seconds	Hold

16 Primers used are as follows:

Component	Sequence
SNP Genotyping assay Forward Primer	TCCTGTTGGGAAACTGACACTTC
SNP Genotyping assay Reverse Primer	TCGGATGGAGCAGTTGTAACG
FAM Probe	ACAGCCCCTGCCCTG
VIC Probe	CAGCCCCCGCCCTG

Citation: gerald.barry , Sharon Flanagan, ÃÂine Rowe, Vivienne Duggan, Erin Markle, Maureen OÃ¢ÂÂBrien PCR protocol to identify an equine mutation associated with Warmblood Fragile Foal Syndrome(WFFS) https://dx.doi.org/10.17504/protocols.io.bw4fpgtn

This is an open access protocol distributed under the terms of the **Creative Commons Attribution License** (https://creativecommons.org/licenses/by/4.0/), which permits unrestricted use, distribution, and reproduction in any medium,
